# Excellent Response to OnabotulinumtoxinA: Different Definitions, Different Predictors

**DOI:** 10.3390/ijerph191710975

**Published:** 2022-09-02

**Authors:** Raffaele Ornello, Carlo Baraldi, Fayyaz Ahmed, Andrea Negro, Anna Maria Miscio, Antonio Santoro, Alicia Alpuente, Antonio Russo, Marcello Silvestro, Sabina Cevoli, Nicoletta Brunelli, Fabrizio Vernieri, Licia Grazzi, Luca Pani, Anna Andreou, Giorgio Lambru, Ilaria Frattale, Katharina Kamm, Ruth Ruscheweyh, Marco Russo, Paola Torelli, Elena Filatova, Nina Latysheva, Anna Gryglas-Dworak, Marcin Straburzyński, Calogera Butera, Bruno Colombo, Massimo Filippi, Patricia Pozo-Rosich, Paolo Martelletti, Simona Guerzoni, Simona Sacco

**Affiliations:** 1 Neuroscience Section, Department of Applied Clinical Sciences and Biotechnology, University of L’Aquila, Via Vetoio 1 Coppito, 67100 L’Aquila, Italy; 2 Digital and Predictive Medicine, Pharmacology and Clinical Metabolic Toxicology-Headache Center and Drug Abuse-Laboratory of Clinical Pharmacology and Pharmacogenomics, Department of Specialist Medicines, AOU Policlinico di Modena, 41125 Modena, Italy; 3 Department of Neurosciences, Hull University Teaching Hospitals, Hull HU3 2JZ, UK; 4 Department of Clinical and Molecular Medicine, Faculty of Medicine and Psychology, Sant’Andrea Hospital, Sapienza University, 00189 Rome, Italy; 5 Headache Center, Unit of Neurology, Fondazione IRCCS “Casa Sollievo Della Sofferenza”, 71013 San Giovanni Rotondo, Italy; 6 Headache Unit, Department of Neurology, Vall D’Hebron University, 08035 Barcelona, Spain; 7 Headache and Neurological Pain Research Group, Vall D’Hebron Institute of Research (VHIR), Department of Medicine, Universitat Autonoma de Barcelona, 08193 Barcelona, Spain; 8 Headache Center, Department of Medical, Surgical, Neurological, Metabolic and Aging Sciences, University of Campania “Luigi Vanvitelli”, 81100 Naples, Italy; 9 IRCCS Istituto delle Scienze Neurologiche di Bologna, 40139 Bologna, Italy; 10 Headache and Neurosonology Unit, Campus Bio-Medico University Hospital, 00128 Rome, Italy; 11 Headache Center, Neuroalgology Department, IRCCS Foundation “Carlo Besta” Neurological Institute, Via Celoria, 11, 20133 Milan, Italy; 12 Pharmacology Unit, Department of Biomedical, Metabolic and Neural Sciences, University of Modena and Reggio Emilia, 41121 Modena, Italy; 13 Department of Psychiatry and Behavioral Sciences, University of Miami, Coral Gables, FL 33146, USA; 14 VeraSci, Durham, NC 27707, USA; 15 Headache Service, Guy’s and St Thomas’ NHS Foundation Trust, London SE1 7EH, UK; 16 Child Neurology and Psychiatry Unit, Systems Medicine Department, Tor Vergata University, 00133 Rome, Italy; 17 Department of Neurology, Ludwig Maximilians University München, 80539 Munich, Germany; 18 Headache Center, Neurology Unit, Neuromotor and Rehabilitation Department, Azienda USL-IRCCS Di Reggio Emilia, 42122 Reggio Emilia, Italy; 19 Headache Center, University of Parma, 43121 Parma, Italy; 20 Department of Neurology, Institute for Postgraduate Education, Sechenov First Moscow State Medical University (Sechenov University), 119435 Moscow, Russia; 21 Headache Center Wroclaw, 50-307 Wrocław, Poland; 22 Department of Family Medicine and Infectious Diseases, University of Warmia and Mazury, 10-719 Olsztyn, Poland; 23 Neurophysiology Service, IRCCS San Raffaele Scientific Institute, 71013 Milan, Italy; 24 Neurology Unit, IRCCS San Raffaele Scientific Institute, 71013 Milan, Italy; 25 Neurorehabilitation Unit, IRCCS San Raffaele Scientific Institute, 71013 Milan, Italy

**Keywords:** chronic migraine, onabotulinumtoxinA, predictors of response, excellent responders

## Abstract

The identification of patients who can benefit the most from the available preventive treatments is important in chronic migraine. We explored the rate of excellent responders to onabotulinumtoxinA in a multicenter European study and explored the predictors of such response, according to different definitions. A pooled analysis on chronic migraineurs treated with onabotulinumtoxinA and followed-up for, at least, 9 months was performed. Excellent responders were defined either as patients with a ≥75% decrease in monthly headache days (percent-based excellent responders) or as patients with <4 monthly headache days (frequency-based excellent responders). The characteristics of excellent responders at the baseline were compared with the ones of patients with a <30% decrease in monthly headache days. Percent-based excellent responders represented about 10% of the sample, whilst frequency-based excellent responders were about 5% of the sample. Compared with non-responders, percent-based excellent responders had a higher prevalence of medication overuse and a higher excellent response rate even after the 1st and the 2nd injection. Females were less like to be frequency-based excellent responders. Chronic migraine sufferers without medication overuse and of female sex may find fewer benefits with onabotulinumtoxinA. Additionally, the excellent response status is identifiable after the first cycle.

## 1. Introduction

Migraine is characterized by unilateral attacks of throbbing, pulsating headache, associated with nausea and vomiting, phonophobia and/or photophobia [1]. Chronic migraine (CM) is characterized by a headache occurring on ≥15 days per month for ≥3 months, displaying migraine features on ≥8 days per month [1]. CM affects about 2% of the general population, affecting especially middle-aged adults [2], thus imposing a significant burden on society [3]. Since this, the development of effective and safe treatment for CM is needed. Before the advent of the monoclonal antibodies (mAbs) acting on the calcitonin gene-related peptide (CGRP) or its receptor, the only drug specifically approved to prevent CM was onabotulinumtoxinA (BT-A) [4]. A large proportion of patients with CM, up to 70% within the first three cycles, respond well to BT-A with a ≥50% reduction in monthly headache days compared with the baseline [5]; however, the rate of ≥50% responders from real-life studies seem lower in the first 3 cycles (49.3%) [6], but a response to the BT-A may be attained even after 12 months—i.e., 5 treatment cycles—from treatment start [7]. Conversely, monoclonal antibodies targeting the CGRP pathway have a quick onset of action, even in CM [8,9,10,11,12,13,14,15]. Therefore, to better understand the place of BT-A in migraine prevention, it would be important to clearly identify patients’ clinical features potentially predicting a substantial response to the drug. An interesting population is that of patients with ≥75% decrease in monthly headache days compared with the baseline, i.e., excellent responders. Previous studies dealt with the determination of the predictors of response to BT-A, according to different definitions, but with conflicting results [16,17,18,19,20,21,22,23,24,25]. The main limitation of the current literature is that the excellent responders are a limited proportion of the treated patients; hence, large collaborations are needed to report reliable results. Furthermore, another possible definition of “excellent responders”, based on the absolute number of residual headache days, could be explored. Indeed, even a patient who reports a ≥75% decrease in monthly headache days could still have a great impairment, especially if the number of headache days at the baseline was very high, thus presenting the same limitations of the 50% of responders [25]. 

The aim of this study was to report the rate of excellent responders according to the percent decrease in headache frequency (percent-based) and a residual headache frequency (frequency-based) in a large European sample after 3, 6 and 9 months, corresponding to the first three cycles of BT-A treatment. Additionally, the rate of patients reporting no headache days at all, i.e., total responders, was also explored. Finally, the potential predictors of an excellent response to BT-A, according to the different definitions, were explored.

## 2. Materials and Methods

### 2.1. Study Design

The methods of this study have been previously published, since two more articles were derived from the analysis of the present dataset [26,27]; this is a pooled patient-level analysis of data from real-life studies on patients with CM treated with BT-A at 16 European headache centers. The centers potentially participating in the study were first identified through a MEDLINE search of the most recent publications on BT-A treatment for CM in Europe. Globally, 19 centers were initially selected and contacted by e-mail. Among them, two centers did not meet the inclusion criteria and one declined to participate in the study. A list of the headache centers participating in this study is available in supplemental Table S1. All centers have already performed, between 2010 and 2020, a real-life prospective study on patients with CM, diagnosed according to the International Classification of Headache Disorders 3rd edition (ICHD-3) criteria [1], aged  ≥ 18 years and treated with BT-A according to the Phase 3 REsearch Evaluating Migraine Prophylaxis Therapy (PREEMPT) protocol [28,29]. Additionally, involved centers must:

1. Have obtained study approval by the local ethics committees and informed consent obtained from patients, if necessary, according to the local regulations;
2. Be able to share a database of collected data;
3. Have planned a follow-up of ≥9 months for all patients, irrespective of the treatment discontinuation.

Since all included centers followed the EHF guidelines to initiate BT-A in CM [30], there were no differences regarding its prescription.

The present analysis was approved by the Internal Review Board of the University of L’Aquila with protocol number 23/2020; approval was shared with all centers; moreover, local ethical approval to pool data was obtained from the centers, if needed. Patients did not need sign any additional informed consent for this study since no additional data with respect to those originally collected were required for the present analyses. All procedures were conducted in accordance with the latest version of the declaration of Helsinki. 

### 2.2. Variables and Response Categories

The following variables were collected: age, sex, duration of migraine, duration of CM, presence of medication overuse (MO), duration of the MO, the mean number of monthly headache days (MHDs) in the last 3 months, the mean number of days per month in which patient took, at least, one medication (MDs) in the last 3 months, other preventive treatments co-assumed among BT-A. Regarding the last point, patients were allowed to continue, at a stable dose, the preventive treatment they were already taking when they started the BT-A. Hence, the effect of the preventative treatment used in combination with BT-A was not quantifiable. Response categories were defined by the percent reduction in mean MHDs during the course of each 3-months treatment cycle compared to the baseline, i.e., the mean of the three months before starting BT-A treatment. Patients who reported 0 headache days during one cycle were indicated as 100% responders, i.e., total responders. Percent-based excellent response was defined as a ≥75% decrease in MHDs from the baseline, as already done in other articles [19,23]. Frequency-based excellent response was defined as a mean of <4 MHDs during the third BT-A cycle, since under 4 MHDs over a 3-months period, the preventive treatment should not be prescribed [31]. Non-response was defined as patients reporting a <30% decrease in MHDs from the baseline [20]. The above-mentioned categories were calculated for every injection cycle, but the comparisons between the different class of responders were performed during the third BT-A cycle, since it is the temporal limit to stop BT-A in case of a poor response [7]. Patients discontinuing treatment before the third cycle or lost at follow-up were considered as non-responders, except if the discontinuation was due to the “positive stopping rule”, i.e., patients reporting a ≥50% response and choosing to withdraw BT-A before the third cycle; these data were included in the analysis using a “last observation carried forward” approach. 

### 2.3. Statistical Analysis

Continuous variables were reported as mean ± standard deviation (SD), whilst categorical variables were reported as counts and percentages. Baseline features were compared between percent-based excellent responders and non-responders. The same calculations were made between frequency-based excellent responders and non-responders. A multivariate logistic model with backward elimination was performed with the variables significantly associated with the response status at the univariate analysis, in order to assess independent predictors of excellent response. The model was then checked for collinearity using the phi-correlation coefficient and the overall goodness of fit of the model was explored with the Pearson’s χ^2^ goodness of fit test. Receiving Operating Characteristic (ROC) curve analysis was used to explore the sensitivity and specificity of those parameters significantly associated with the responder status even in the multivariate analysis. All analyses used the intent-to-treat population, including all patients who started BT-A treatment, but variables were considered for the analyses only if available for, at least, two-thirds of patients. No imputation was done for missing data. Since the calculations were made on available data, no sample size calculations were made. P-values lower than 0.05 (two-tailed) were considered significant. All calculations were made with STATA IC 13.1 software.

## 3. Results

### 3.1. Baseline Features

Globally, data from 2879 patients were included, of whom 2351 (81.7%) were women. The mean age was 46.6 ± 12.32 years, whilst the mean migraine duration was 30.74 ± 12.84 years. Patients with MO were 2055 (71.4%). At the baseline, patients displayed MHDs of 23.82 ± 5.93 and a mean number of MDs of 19.47 ± 9.05; these data are summarized in Table 1.

### 3.2. Rates of Excellent Responders and Total Responders

**Figure 1:**
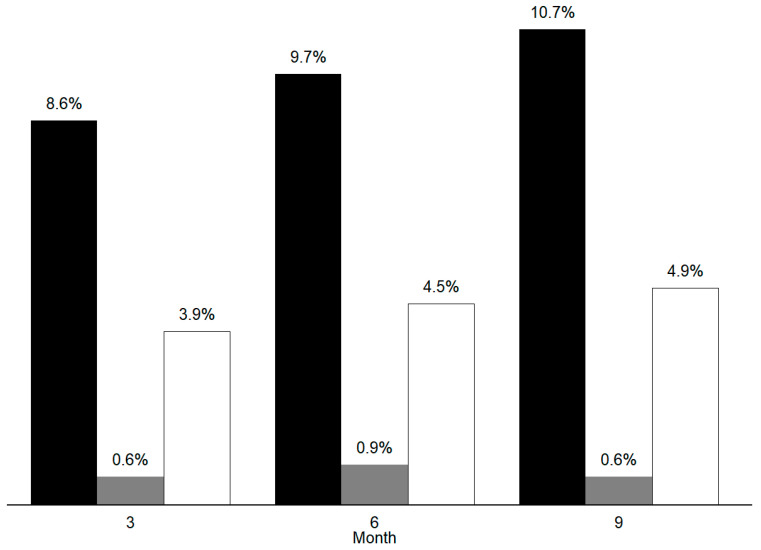
Overall number of percent-based excellent responders to onabotulinumtoxinA (black bars), the overall number of total responders (grey bars) and the overall number of the frequency-based excellent responders (white bars) during the first, second, and third onabotulinumtoxinA cycle.

Total responders were 18 (0.6%) during the first, 26 (0.9%) during the second, and 18 (0.6%) during the third treatment cycle. Percent-based excellent responders were 248 (8.6%) during the first, 278 (9.7%) during the second, and 307 (10.7%) during the third treatment cycle. Considering any of the three BT-A cycles, 532 (18.5%) patients were classifiable as percent-based excellent responders. Eighty-four patients (2.9%) were classifiable as percent-based excellent responders during all three treatment cycles. Frequency-based excellent responders were 112 (3.9%) during the first, 130 (4.5%) during the second, and 140 (4.9%) during the third BT-A cycle. Frequency-based excellent responders in, at least, one cycle were 272 (9.5%), whilst 22 patients (0.76%) were classifiable as frequency-based excellent responders in all injection cycles. Non-responders were 1564 (54.32%) during the first, 1374 (47.72%) during the second, and 1376 (47.79%) during the third treatment cycle. Data regarding percent-based excellent responders, total responders and frequency-based excellent responders are graphically summarized in Figure 1.

### 3.3. Comparison between Percent-Based Excellent Responders and Non-Responders

Compared with non-responders, percent-based excellent responders displayed a higher prevalence of MO (71.7% vs. 63.8%, *p* = 0.001) and a higher medication consumption at the baseline (20.97 ± 9.45 vs. 18.10 ± 9.95, *p* < 0.001); moreover, percent-based excellent responders at the 9th month displayed a higher proportion of excellent responders even at the 3rd (52.85% vs. 47.15%, *p* < 0.001) and the 6th months (77.83% vs. 22.17%, *p* < 0.001). At the multivariate analysis, the presence of MO at the baseline and an excellent response during the first and the second BT-A cycles were significantly associated with percent-based excellent response during the third BT-A cycle. these data are summarized in Table 2. The Pearson’s χ^2^ test displayed a general goodness of fit of the entire model (Pearson’s χ^2^ = 509.6, *p* = 0.0093). The ROC curve analysis displayed an area under the curve of 0.7813.

### 3.4. Comparison between Frequency-Based Excellent Responders and Non-Responders during the Third BT-A Cycle 

At the third injection cycle, only the female sex resulted significantly associated with the frequency-based excellent responders, whilst none of the other variables was. In particular, the proportion of females was significantly lower in the frequency-based excellent responders. These data are summarized in Table 3.

## 4. Discussion

According to the present study, the rate of percent-based excellent responders to BT-A was about 10% in every injection cycle and about 3% of patients were classifiable as percent-based excellent responders in all cycles. In a previous Spanish study, the proportion of percent-based excellent responders to BT-A was about 20% after 6 months and 25% after one year [20]; the higher number of HD and MD at baseline in the present sample may account for the observed discrepancies. Total responders were less than 1% during all BT-A cycles. Anyhow, the stability of this outcome suggests that BT-A action lasts in time, as already assessed in the open-label phase of the PREEMPT trial [29]; however, patients obtaining a 50% or even a 75% response compared with the baseline might indeed have a substantial number of days with headache and, consequently, a high headache-related disability: many patients would still have >8 debilitating HDs per month, configuring resistance to BT-A [6,25]. The proportion of frequency-based excellent responders was about 4% and, indeed, this criterion is far more restrictive. Our data show that frequency-based criteria identify a lower proportion of responders than percent-based criteria; this finding is in line with what was found in a real-life study on erenumab [26]. The clinical consequence of this finding is that many patients on treatment with BT-A could benefit from combination treatments for the prevention of migraine. Oral treatments are particularly suitable for combination with BT-A as the toxin has a local action without systemic absorption; therefore, it has no interaction with systemic treatments. Identifying patients with CM with an excellent response to BT-A has gained importance with the advent of the effective, safe, and specific mAbs acting on the CGRP pathway [31]; it is increasingly important to select patients who can have a substantial benefit from BT-A in order to better target treatments. In patients with CM in whom an excellent response to BT-A can be anticipated, starting and continuing the treatment is a suitable option, while in patients in whom an excellent response cannot be anticipated, strict monitoring could be advised, with the possibility of switching to other treatments [32], or adding new ones, such as an anti-CGRP mAb [33]. Anyway, the choice of combining 2 preventive treatments if often not supported by well-designed randomized-controlled trials [34] and, consequently, some authors suggest that it should only be cautiously considered only in the case of refractory CM [35]. Response to BT-A is assessed over 2–3 cycles [7], corresponding to 6–9 months, during which patients with CM might still suffer from frequent and debilitating headaches. Response to BT-A can have different definitions [36], each identifying different proportions of patients [37]. Excellent responders are an interesting group of patients, as they represent those who can continue treatment with BT-A without starting concurrent or alternative preventive treatments. The absolute number of headache days is, in our opinion, a more reliable way to quantify the benefit of migraine prevention; patients with <4 headache days per month do not require further migraine prevention according to common practice guidelines [31]. Notably, both groups of excellent responders may be distinguished from non-responders according to the baseline characteristics. The present study found that the presence of MO and the excellent responder status at 3 and 6 months were independent predictors of the percent-based excellent responder status in comparison with non-responders (Table 2). Unexpectedly, the proportion of MO sufferers at the baseline was higher in excellent responders than non-responders whilst, in other studies, the presence of MO was negatively associated with the BT-A response [20,24]. Data from animal models identify MO as a risk factor for peripheral sensitization of the trigeminal pathway [38], since an up-regulation of CGRP in the trigeminal ganglion has been found in rats [39]. In humans, CGRP has been linked with trigeminal-mediated pain [40] and its levels have been found higher in the peripheral blood of CM and MO sufferers [41]; moreover, BT-A has been found to decrease inter-ictal levels of CGRP in CM sufferers [42], thus making CGRP plasma levels as possible predictors of response to BT-A [17,19]; thus, patients with MO should be characterized by a higher release of CGRP by trigeminal C-fibers, which is blocked by BT-A [43]. Indeed, BT-A inhibits the SNARE complex and, therefore, blocks the exocytosis of CGRP from trigeminal C-fibers [43]. In this context, patients who suffer from MO and, hence, who have a higher CGRP release, should find higher benefits with BT-A; it should be noted that the significance of MO in the model was marginal and could be prone to bias. The absolute difference in MDs and in the prevalence of MO between excellent responders and non-responders was relatively low; therefore, we cannot exclude a spurious significance, even because of the high number of patients which could have increased the risk of falsely significant results. Another notable finding of our paper is that the early status of excellent responder was the strongest predictor of excellent response during the third BT-A cycle (Table 2); this finding is in line with a previous publication on the same subset which showed that BT-A response status during the second BT-A cycle could predict response during the third cycle [7]. Therefore, even when considering an excellent response to BT-A, it might not be necessary to wait for the third BT-A cycle to plan a switch to other migraine preventatives; this is a relevant finding in the era of anti-CGRP treatments [32].

The excellent responders at the 9th month displayed a higher proportion of excellent responders even at the 3rd and the 6th month; this suggests that the excellent response can be distinguished from a non-response even from the first injection cycle and, again, this witness that BT-A spreads rapidly [44] and that action lasts on time [45]. The HD and the MD were significantly associated with the responder status, but only at the univariate analysis and, consequently, they are not considerable as independent predictors of an excellent response versus non-response. Despite this, a large body of literature revealed that migraine frequency at the baseline was negatively associated with BT-A response [18,24]. Interestingly, our results partially confirm the ones obtained in a previous study from Pozo-Rosich’s group [24]. In that study, the factors independently associated with a percent-based excellent response at 6 months were daily headache frequency, the presence of MOH, and a higher number of HD at the baseline. The duration of migraine was found to be associated with an excellent response to the BT-A, but after 12 months [24]. The HD was also found to be associated with a poor response to BT-A in a study by Dominguez et al. [21] and our study seems to confirm this association. In particular, a higher number of HD at the baseline may negatively predict the response to BT-A compared to poor-responders; this suggests that a greater burden of CM at baseline may account for a lower response after 9 months of BT-A of treatment, in line with the available literature [18,20,22]. Again, the excellent responders’ group in the ninth month showed a higher number of excellent responders in the 6th month of treatment, confirming that the effect of the BT-A spreads in the first months [44] and is almost stable on time. The frequency-based excellent responders displayed a lower proportion of females at the baseline. The gender difference in BT-A response has been studied, but with conflicting results. In particular, a previous study failed to find a clear gender difference in the clinical response towards BT-A [27]. Despite its statistical significance, sex difference in the response towards BT-A is gravated by the great difference between men and women numerosity as well as a higher number of drop-outs among men; thus, the exact magnitude of this difference is difficultly detectable but, at least, questionable. Globally, this study allowed the description of a limited subgroup of patients with CM and an excellent response to BT-A in a large group of almost 3000 patients treated in clinical practice; this number ensures the reliability of our findings even if regarding a limited proportion of patients. Besides, we could analyze two different definitions of excellent response to BT-A, one based on traditional percent-based response status and one based on absolute headache frequency, which could be clinically significant; however, our study also has some limitations. We could not quantify the effect of the preventative treatment used in combination with BT-A, which was allowed as the present study was based on clinical practice. Although the data were collected prospectively, the study had a there was significant heterogeneity of data collection across centers. We minimized the collected data in order to limit missing information and inaccuracy; however, this led to missing important information, including the presence of cutaneous allodynia, the number of prior preventive treatment failures, BT-A dosage, and a number of medical comorbidities which may have affected response to BT-A [22,24,45]. A further study limitation is represented by the heterogeneity in the quantitative contribution of different centers, since large centers might have had a greater impact on the study than the smaller ones. The number of drop-outs may have also led to the underestimation of excellent responders as some of them stopped the treatment according to a “positive stopping rule” [46]. Furthermore, we did not distinguish migraine days from headache days, which could have added further detail to the outcome assessment. Lastly, we could not distinguish patients discontinuing BT-A because of ineffectiveness from those who discontinued because of non-compliance.

## 5. Conclusions

The present study suggested that the proportion of sustained percent-based excellent responders to BT-A is about 10% during the first three injection cycles, while the proportion of sustained frequency-based excellent responders is about 3%. Early excellent response to BT-A predicted response during the third BT-A cycle, which is a relevant finding when planning treatment strategies in clinical practice. Factors independently associated with percent-based excellent response included the presence of MO and a lower number of headache days per month at baseline. In brief, our data indicate that the more the impairment of patients at the baseline is, the less the therapeutic benefit with BT-A. 

## Figures and Tables

**Table 1:** Baseline characteristics of the analyzed sample.

Variable	Value
Number of patients	2879
Age (years)	46.6 ± 12.3
Female sex	2351 (81.7)
Migraine duration (years)	30.7 ± 12.8
CM duration (years)	8.0 ± 8.0
Medication overuse at baseline	2055 (71.38)
MHDs	23.8 ± 5.9
MDs	19.4 ± 9.1

**Table 2:** Comparison between the percent-based excellent responders and the non-responders after the third BT-A cycle.

	*Status at the 9th Month*		*Univariate Analysis*		*Multivariate Analysis*	
*Baseline Characteristics*	*Excellent* *Responders* *(n = 307)*	*Non-Responders* *(n = 1376)*	*p-Value*	*OR (95% CI)*	*p-Value*	*OR (95% CI)*
Age	46.68 ± 12.55	46.79 ± 12.89	0.887	-	-	-
Female sex	241/307 (%)	1101/1376 (%)	0.604	0.92 [0.68 ÷ 1.25]	-	-
Migraine duration	28.20 ± 12.77	30.63 ± 13.74	0.056	-	-	-
CM duration	8.66 ± 9.27	8.34 ± 8.80	0.603	-	-	-
**MO**	**220/307 (71.66%)**	**878/1376 (63.81%)**	**0.001**	**1.59 [1.20 ÷ 2.12]**	**0.046**	**1.91 [1.01 ÷ 3.62]**
Headache days	25.71 ± 5.38	24.42 ± 6.16	0.001	-	0.106	1.02 [0.99 ÷ 1.06]
Medication days	21.20 ± 9.58	18.10 ± 9.95	<0.001	-	0.877	1 [0.97 ÷ 1.03]
**Excellent responders et the 3rd month**	**102/307 (52.85%)**	**91/1376 (47.15%)**	**<0.001**	**1.95 [1.63 ÷ 2.27]**	**0.047**	**1.63 [1.01 ÷ 2.64]**
**Excellent responders et the 6th month**	**158/307 (77.83%)**	**45/1376 (22.17%)**	**<0.001**	**3.45 [3.07 ÷ 3.82]**	**<0.001**	**22.14 [14.37 ÷ 34.10]**

Abbreviations: CM: chronic migraine; MO: medication overuse.

**Table 3:** Comparison between frequency-based excellent responders and non-responders during the third onabotulinumtoxinA cycle.

			*Univariate Analysis*		*Multivariate Analysis*	
*Baseline Characteristics*	*Frequency-Based Excellent Responders (n = 140)*	*Non-Responders (n = 1376)*	*p-Value*	*OR (95% CI)*	*p-Value*	*OR (95% CI)*
Age	46.1 ± 12.9	46.79 ± 12.9	0.52	-	Not applicable	
**Female sex**	**101/140 (72.1%)**	**1101/1376 (80%)**	**0.041**	**0.71 [0.41 ÷ 0.98]**		
Migraine duration	28.8 ± 13.9	30.6 ± 13.7	0.345	-		
CM duration	8.6 ± 8.3	8.3 ± 8.8	0.715	-		
MO	68.6%	65.2%	0.103	1.39 [0.93 ÷ 2.06]		
Headache Days	23.4 ± 6.6	24.4 ± 6.2	0.064	-		
Medication Days	19.7 ± 9.5	18.1 ± 9.9	0.104	-		

Abbreviations: CM: chronic migraine; MO: medication overuse headache.

## Data Availability

The dataset analyzed during this study is available from the corresponding author on reasonable request.

## Supplementary Materials

The following supporting information can be downloaded at: https://www.mdpi.com/article/10.3390/ijerph191710975/s1, Table S1: Overview of the study centers.

## Abbreviations

BT-A	onabotulinumtoxinA
CGRP	calcitonin gene-related peptide
CM	chronic migraine
CSD	cortical spreading depression
HIT-6	6 items headache impact test
ICHD-3	International Classification of Headache Disorders 3rd edition
mAbs	monoclonal antibodies
MIDAS	Migraine Disability Assessment Questionnaire
MHDs	the mean number of monthly headache days in the last 3 months
MDs	the mean number of days per month in which patient took at least one medication (MDs) in the last 3 months
MO	medication overuse
PREEMPT	Phase 3 REsearch Evaluating Migraine Prophylaxis Therapy
ROC	Receiving Operating Characteristic
SD	standard deviation
