# Supplementary material for: Excellent Response to OnabotulinumtoxinA: Different Definitions, Different Predictors
Source: Int J Environ Res Public Health. 2022 Sep 2;19(17):10975. doi: 10.3390/ijerph191710975 (PMC9518492; doi:10.3390/ijerph191710975)
Supplement: Supplementary file 1 [file ijerph-19-10975-s001.zip › ijerph-1860111-supplementary.pdf]

Table S1. Overview of the study centers.

Center	No. (%) of patients included	Inclusion period	Stopped treatment or lost to follow-up at 3 months (n, %)	Stopped treatment or lost to follow-up at 6 months (n, %)	Stopped treatment or lost to follow-up at 9 months (n, %)
Hull	707 (24.6)	July 2010 – Mar 2020	2 (0.3)	256 (36.2)	384 (54.3)
Rome (Sant'Andrea)	564 (19.6)	Jan 2017 – Mar 2020	1 (0.2)	8 (1.4)	17 (3.0)
San Giovanni Rotondo	455 (15.8)	Mar 2013 – Mar 2020	60 (13.2)	117 (25.7)	147 (32.3)
Barcelona (Vall d'Hebron)	248 (8.6)	July 2014 – Sep 2018	0	54 (21.8)	85 (34.3)
Milan (San Raffaele)	159 (5.5)	May 2015 – May 2020	3 (1.9)	10 (6.3)	32 (20.1)
Naples (Vanvitelli)	107 (3.7)	Feb 2015 – Jul 2020	0	0	0
Bologna	102 (3.5)	Jan 2014 – May 2020	1 (1.0)	3 (2.9)	34 (33.3)
Rome (Campus Biomedico)	92 (3.2)	Sep 2013 – Apr 2020	0	0	2 (2.2)
Milan (Besta)	78 (2.7)	Oct 2017 – Oct 2019	3 (3.8)	8 (10.3)	11 (14.1)
Modena	75 (2.6)	Jan 2015 – Jan 2020	0	0	0
London (St Thomas')	70 (2.4)	Jan 2019 – Sep 2020	0	2 (2.0)	15 (21.4)
Moscow (Sechenov)	53 (1.8)	Jan 2018 – Mar 2020	0	9 (5.2)	12 (22.6)
L'Aquila	52 (1.8)	Dec 2016 – July 2018	0	0	0
Munich	48 (1.7)	Dec 2019 – May 2020	0	6 (12.5)	17 (35.4)
Parma	45 (1.6)	Feb 2014 – Apr 2016	0	19 (42.2)	30 (66.7)
Wroclaw	24 (0.8)	Nov 2017 – Jun 2020	3 (12.5)	4 (16.7)	13 (54.2)
TOTAL	2879		73 (2.5)	496 (17.2)	799 (27.8)
